# Thymol Nanopolymer Synthesis and Its Effects on Morphine Withdrawal Syndrome in Comparison With Clonidine in Rats

**DOI:** 10.3389/fnbeh.2022.843951

**Published:** 2022-06-29

**Authors:** Ardeshir Moayeri, Reza Mehdizadeh, Elahe Karimi, Ali Aidy, Hori Ghaneialvar, Naser Abbasi

**Affiliations:** ^1^Department of Anatomy, Medical School, Ilam University of Medical Sciences, Ilam, Iran; ^2^Biotechnology and Medicinal Plants Research Center, Ilam University of Medical Sciences, Ilam, Iran; ^3^Department of Pharmacology, Medical School, Ilam University of Medical Sciences, Ilam, Iran

**Keywords:** nano polymer, thymol, *Thymbra spicata*, morphine, withdrawal syndrome

## Abstract

The drug delivery system is valuable in the treatment of the disease. A nanopolymer as a thymol and *Thymbra spicata* release system was synthesized and its effects on morphine withdrawal syndrome in comparison with clonidine in rats were studied. The nanopolymer was characterized by different methods, namely, IR, HNMR, CNMR, GPC, DLS, and AFM. Thymol in *T. spicata* extract was assessed. The loading and release rate of thymol and *T. spicata* extract on the nanopolymer were evaluated by HPLC. The median lethal dose (LD_50_) of the *T. spicata* extract, thymol, extract nanopolymer, and thymol nanopolymer was studied. The frequency of jumping, rearing, and teeth chattering in naloxone-induced morphine withdrawal syndrome was studied. Synthesized nanopolymer was desirable as a carrier for the drug. The loaded amount of extract and thymol on nanopolymer was estimated 55 ± 3.2% and 48 ± 2.6% and the drug released was 71 and 68%, respectively. LD_50_ of the *T. spicata* extract, thymol, extract nanopolymer, and thymol nanopolymer was 975, 580, 1,250, and 650 mg/kg, respectively. This study showed that thymol nanopolymer was more effective than clonidine to reduce the frequency of morphine withdrawal symptoms. Our results suggest that *T. spicata* extract, thymol, extract nanopolymer, and thymol nanopolymer are mighty in reducing the narcotic withdrawal signs. The mechanism of action and therapeutic potential is maybe similar to clonidine.

## Introduction

Many benefits, namely, improved drug solubility, stability, reduced clearance rate, increased drug exposure, and fewer side effects, have been reported for nanoscale drug delivery (Moghimi et al., 2005; Wagner et al., 2006; Kim et al., 2010). The size of the nanoparticles carrier plays an important role in the production of nanodrugs to improve biodistribution, tissue penetration, and function (Stano et al., 2012; Tang et al., 2014). The amount of drug-loaded on the nanoparticle depends on factors such as the branches of hydrophobic and hydrophilic polymers (Chu et al., 2013). Citric acid is one of the most inexpensive and compatible substances that is also used in the pharmaceutical and food industries (Naeini et al., 2010). In addition to citric acid, glycerol can be used to synthesize a biocompatible polymer (Lee et al., 2001). Fourier transforms infrared (FTIR), nuclear magnetic resonance (NMR), gel permeation chromatography (GPC), dynamic light scattering (DLS), and atomic force microscopy (AFM) are used to investigate the physicochemical properties of the produced polymeric nanoparticles (Adeli et al., 2013).

Today, dependence on opioids is a world health problem that makes many economic, social, and individual problems. In this regard, the use of nanocarriers loaded with naloxone has been considered in the treatment of opioid overdose (Hasan et al., 2021). Nevertheless, the challenge of using nanoparticles loaded with a bioactive component of medicinal plants remains.

Withdrawal symptoms in morphine addicts are relatively severe and include hypertension, diarrhea and vomiting, and dysphoria (Ballantyne and LaForge, 2007). Mu-, kappa-, and delta-opioid receptor agonists, such as methadone, are effective in helping to quit an addiction with analgesic effects, but there are side effects such as constipation, and respiratory depression, physical dependence, and addiction themselves (Li and Zhang, 2012). However, common pharmacological treatments for withdrawal syndrome include MOR agonists (methadone) or multifunctional opioid/nociception-OFQ agonists (buprenorphine) (Kreek et al., 2002).

Medicinal herbs with a variety of active components have been used potentially in the treatment of withdrawal syndrome (Ward et al., 2011; Tabatabai et al., 2014). Numerous studies have shown that various medicinal plants such as *Passiflora incarnate*, *Hypericum perforatu*, *Avena sativa*, and *Valeriana officinalis*, with their antianxiety, hypnotic, analgesic, and antispasmodic effects, have improved the symptoms of withdrawal syndrome (Ebrahimie et al., 2015). Although there was no evidence of the morphine withdrawal and anti-anxiety effect of *Thymbra spicata* extract in the previous studies, *T. capitata* (L.) extract of the same family had a significant antianxiety effect (Motti and de Falco, 2021). The anti-analgesic and antinociceptive effects of the *Thymus vulgaris L*. extract have also been documented (Rašković et al., 2020). Many medicinal plant oils such as *T. spicata*, *Thymus vulgaris*, *Monarda fistulosa*, *Trachyspermum ammi*, *Thymus ciliates*, and *Nigella sativa*, contain a monoterpene named thymol (Wattanasatcha et al., 2012), which can be used as a food additive under the Food and Drug Administration regulations (Dhaneshwar et al., 2013). Due to the chemical structure of thymol, it can be used to produce nanoparticles to treat various diseases (Jafari et al., 2018; Robledo et al., 2018). Thymol has previously been shown to attenuate the signs and symptoms of an opiate withdrawal syndrome in humans (Khodayar et al., 2014). Thymol not only has been able to reduce the anxiety associated with diazepam withdrawal but also improves motor and memory impairment in rats (Saleem et al., 2021). In this study, in addition to synthesizing a nanopolymer and binding it with thymol, the effects and possible mechanism on morphine withdrawal syndrome in rats were investigated.

## Materials and Methods

### Preparation of Nanopolymer

By melt polycondensation method, citric acid and glycerol (Carlo Erba) for the synthesis of branched polymer, were combined and dissolved in tetrahydrofuran (THF) (Merck) and filtered. The solution was precipitated in cyclohexane (Merck) and dissolved in THF and then placed in a dialysis bag (Mn cutoff 2000, Sigma-Aldrich) (Adeli et al., 2013). Then, oleic acid was poured into a polymerization capsule and heated at 90, 100, 120, 140, and 160°C, respectively.

### Fourier Transforms Infrared Spectroscopy

The nanopolymer IR spectra were performed with an FT–IR (Nicolet 320 spectrophotometer). The spectra were obtained at a resolution of 4 cm^–1^ in the range 4,000–500 cm^–1^ (Dong and Gu, 2002).

### Nuclear Magnetic Resonance

Identical NMR spectra were obtained by dissolving samples in D_2_O (Bruker DRX 400 MHz) and the spectra were recorded at 500 MHz. The resulting data were analyzed using ACDLABS/1D NMR software (Neri et al., 1989).

### Gel Permeation Chromatography

Using Gel permeation chromatography (Knauer equipped with Smartline Pump 1,000 with an PL Aqua gel-OH mixed-H 8 μm column), molecular weights and distribution of the obtained nanopolymer were determined (Lee, 1978).

### Dynamic Light Scattering

Dynamic light scattering data (Malvern Instruments Ltd., United Kingdom were measured in water three times (5 runs to each measurement). The mean size was accounted as the six measurements average (Brown et al., 1992).

### Atomic Force Microscopy

A multimode scanning probe microscope (Ara-research Inc., Iran) for atomic force microscopy (AFM) was used and the nanopolymer morphology was determined. The nanopolymer suspension (a droplet) was dried by a freeze dryer (Christ, Germany) onto a clean surface of mica before AFM imaging. The size of the images was 5 × 5 μm. The images were scanned on at least six different areas of the sample (Rezayati Charani et al., 2013).

### Thymol Identification by High-Performance Liquid Chromatography

The aerial parts of *Thyme spicata* were collected from Ilam, Iran, and identity was authenticated by the voucher specimens (NO. 596) deposited in the Department of Horticulture, Faculty of Agriculture, Ilam University. After drying and powdering, the powder was degreased by hexane and 20 g of the plant was used for extraction by a Soxhlet extraction method in a water–ethanol solvent. The plant powder was loaded into the porous cellulose thimble (20 g in a 25- × 80-mm thimble), which was placed inside the Soxhlet extractor. The sidearm was lagged with glass wool. The solvent (250 ml of water–ethanol) was heated and evaporated. By rotary (IKA HB 10, Germany) device, the solvents were removed. The extraction (yield = 5.67%) was lyophilized and kept stored at –20°C and were dissolved in methanol (Lopez et al., 2013). According to the previous procedure, the high-performance liquid chromatography (HPLC) method was done (Aghamohammadi et al., 2016). A reversed-phase UV-HPLC (Smart line; Knauer, Germany) with a C18 column (Nucleosil H.P.; 25 cm × 0.46 cm internal diameter, particle size 3 μm, 100 Å pore size) was developed for the thymol determination. The *T. spicata* extract peaks were compared with the thymol standard (Sigma Aldrich). A stock solution (0.1 mg/ml) of thymol standard diluted to obtain 15.6, 31.25, 62.5, 125, 250, and 500 μM (Moayeri et al., 2018).

### Thymol Encapsulation

Nanopolymer (0.1 g, 1.67 × 10^–2^ mM) was dissolved in distilled water (5 ml) and stirred for 1 h. Then, thymol and extract dissolved in DMSO (Merck) were added dropwise to a nanopolymer mixture and various concentrations (25, 50, 100, and 150 μM) were obtained (Adams et al., 2003).

### Releasing Capacity Assay

Loading capacity was determined by the HPLC method (Lv et al., 2009) and estimate the amount of thymol and extract loaded on the nanopolymer. The mobile phase was 40% methanol and 60% aqueous solution of formic acid (0.1%) (Majumdar et al., 2014). The solution aliquot was injected into the HPLC.

*In vitro* release of thymol and extract from nanopolymer was done by dissolving of thymol (5 mg) and thymol in extract-loaded nanopolymer in PBS (3 ml, 0.1 M, pH 7.4). At the sampling time, the release medium was replaced by a fresh buffer and injected into the HPLC (Qiao et al., 2005).

### Animals

Rats (male albino, 180–260 g, Institute of Medicinal Plants, ACECR, Karaj, Iran) were used. The rats were maintained at standard conditions (22–25°C, 12 h dark-light cycle, standard rodent feed, and water). In total, eight rats were used for each dose of the thymol, extracts, and drugs (*n* = 3). The method grouping to study morphine withdrawal syndrome was as Table 1.

**TABLE 1:** Animals grouping and *Thymbra spicata* extract, thymol, extract nanopolymer, and thymol nanopolymer used to create dependence and morphine withdrawal syndrome.

Group 1	Morphine (control)
Group 2	Morphine and clonidine (positive control)
Group 3	Morphine and extract 100 mg/kg
Group 4	Morphine and extract 200 mg/kg
Group 5	Morphine and extract 300 mg/kg
Group 6	Morphine and Thymol 30 mg/kg
Group 7	Morphine and Thymol 60 mg/kg
Group 8	Morphine and Thymol 90 mg/kg
Group 9	Morphine and extract nanopolymer 100 mg/kg
Group 10	Morphine and extract nanopolymer 200 mg/kg
Group 11	Morphine and extract nanopolymer 300 mg/kg
Group 12	Morphine and Thymol nanopolymer 30 mg/kg
Group 13	Morphine and Thymol nanopolymer 60 mg/kg
Group 14	Morphine and Thymol nanopolymer 90 mg/kg
Group 15	Non-dependent rats (saline and vehicle)

### Ethical Approval

The study was approved by the Ilam University of Medical Sciences, Ilam, Iran. The ethical approval for this study was obtained from the Animal Care and Ethics Committee (ACEC) of the Ilam University of medical science (IR.MEDILAM.REC.1396.81).

### The Median Lethal Dose

To obtain the median lethal dose, different doses of the *T. spicata* extract, thymol, extract nanopolymer, and thymol nanopolymer were administered (orally gavage, 20 rats in each group). The doses were 50, 200, 400, 600, 800, 1,000, 1,600, and 2,400 mg/kg for *T. spicata extract* and extract nanopolymer and 50, 100, 200, 300, 400, 500, 600, and 700 mg/kg for thymol and thymol nanopolymer, respectively. Then, 72 h after gavage, the median of dead animals was calculated. Mortality graphs were plotted between log-concentration vs. percent mortality and the LD_50_ was determined (Parmar and Prakash, 2006; El Kabbaoui et al., 2017). Therefore, the *T. spicata* extract and extract nanopolymer with doses of 100, 200, 300 mg/kg and thymol and thymol nanopolymer with doses of 30, 60, and 90 mg/kg was selected to continue the experiments because none of the used doses caused mortality and were considered in the ED_50_ range.

### Method of Morphine Dependency

Morphine (Darou Pakhsh Pharmaceutical Company, Iran) was injected subcutaneously into the rats two times daily for 7 days. On days 1 and 2, a dose of morphine (2.5 mg/kg) was used two times daily and doubled every day until the day 6, reaching 40 mg/kg. Rats received the last dose (50 mg/kg) on day 7 (Romandini et al., 1984).

### Withdrawal Syndrome Method

In all the groups, 4 h after the last dose of morphine, animals received naloxone (3 mg/kg, i.p., Sigma-Aldrich Company, United States). Immediately, the morphine withdrawal symptoms manifestations, namely, jumping, rearing, and teeth chattering, were monitored for half an hour (Feily and Abbasi, 2009).

Animals of all groups received intraperitoneally 3 mg/kg naloxone 4 h after the last injection of morphine on day 7 of the morphine treatment. Immediately after the naloxone injection, each animal was placed in a transparent acrylic cylinder to observe the frequency of withdrawal manifestations (jumping, rearing, and teeth chattering) for 30 min.

### Coadministration Use of *Thymbra spicata* Extract, Thymol, Extract Nanopolymer, Thymol Nanopolymer on Morphine Withdrawal Syndrome

Rats in groups 3, 4, 5, and 15 received *T. spicata* extract (100, 200, and 300 mg/kg, NG tube), and normal saline, and received thymol at 30, 60, and 90 mg/kg in groups 6, 7, and 8, respectively. Rats in groups 9, 10, and 11 received extract nanopolymer (100, 200, 300 mg/kg, NG tube), and received thymol nanopolymer at 30, 60, and 90 mg/kg in groups 12, 13, and 14, respectively, simultaneous with morphine (two times daily for 6 days). To challenge examine, 4 h before naloxone injection, animals in treatment groups received the last dose on day 70, and group 15 received saline with morphine.

### Method of Coadministration Use of Clonidine Hydrochloride and Morphine on Morphine Dependency and Withdrawal Syndrome

Clonidine hydrochloride (0.2 mg/kg i.p.) was used concomitantly with morphine two times daily (6 days) in group 2. The animals received the last dose on day 7, 4 h before receiving naloxone, to examine jumping, rearing, and teeth chattering.

### Statistical Analysis

The results were shown as mean ± standard error (SEM). One-way ANOVA followed by Tukey *post hoc* test was used for data analysis.

## Results

### Nanopolymer Evaluation

**FIGURE 1:**
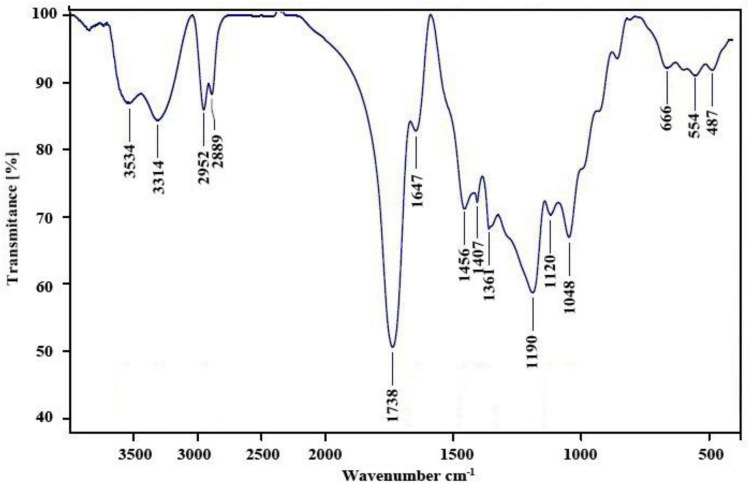
FT-IR spectrum of nanopolymer.

The FT–IR spectrum of the nanopolymer compound was shown (Figure 1). The peak related to the carbonyl ester group, hydroxyl groups, and carbonyl group appears at 1,647, 3,314, and 1,738 cm^–1^, respectively.

**FIGURE 2:**
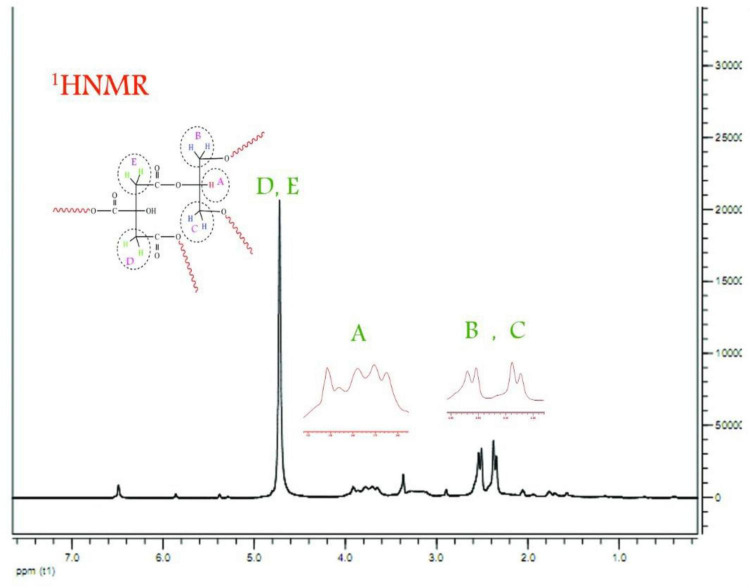
^1^H NMR spectrum of nanopolymer in D_2_O.

The nanopolymer ^1^HNMR spectrum was shown (Figure 2). Hydrogen A was carbonyl group methane in the oleic acid monomer (3.82 ppm). Hydrogen B was monomer glycerol (2.68 ppm). Hydrogen C was methylated glycerol. Hydrogens D and E were the citric acid hydrocarbon content (3.36 ppm).

**FIGURE 3:**
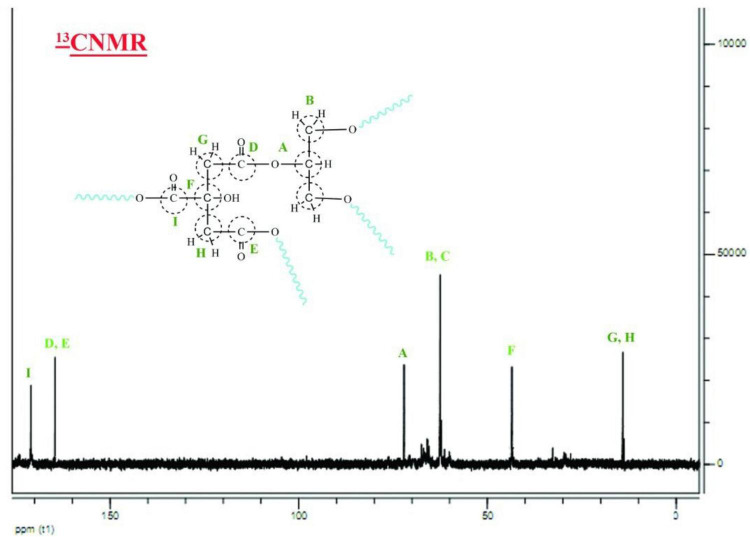
^13^C NMR spectrum of nanopolymer in D_2_O.

The nanopolymer ^13^CNMR spectrum was presented (Figure 3). This combination had a 9-peak index in the carbon compounds of this composition (letters A–I). Carbon A the middle carbon of glycerol (74 ppm). Carbons B and C were methyl glycerol carbonates (68 ppm). Carbons D and E were the two Carboniferous carbon (172 ppm). Carbon F was the fourth type of citric acid (44 ppm). Carbons G and H were the methyl group (13 ppm). Carbon G was the carbonyl oleic acid group (183 ppm).

**FIGURE 4:**
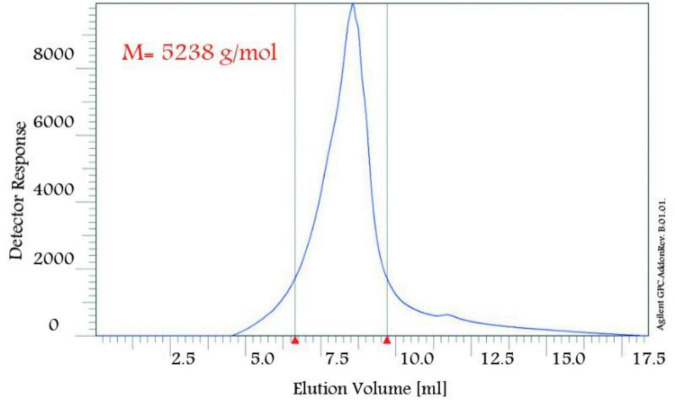
GPC image of nanopolymer.

**FIGURE 5:**
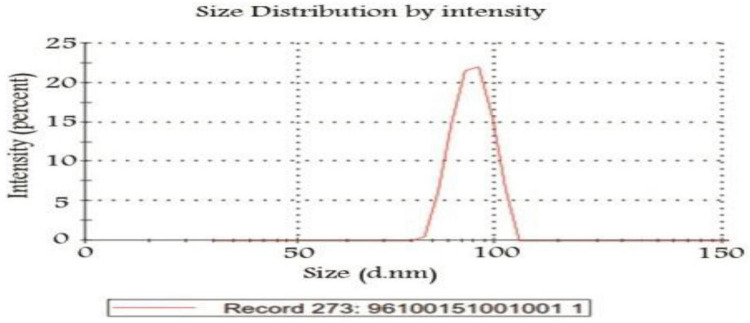
DLS diagram of nanopolymer.

The nanopolymer GPC diagram was shown in Figure 4. This molecular weight was 5,238 g/mol. The size of the nanopolymer was monitored (in water, 25°C) by DLS (Figure 5). The peak width indicated a particle size sample solution larger than 75 nm, but the diameter of most particles was estimated to be about 100 nm.

The size of the nanopolymer was monitored (in water, 25°C) by DLS (Figure 5). The peak width indicated a particle size sample solution larger than 75 nm, but the diameter of most particles was estimated to be about 100 nm.

**FIGURE 6:**
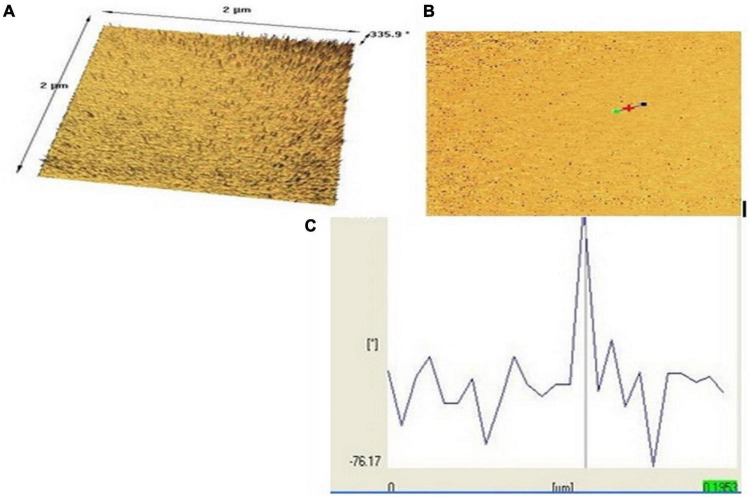
Nanopolymer AFM images of surface morphology [top (A), bottom (B), and (C) topographical view]. (*) has been done by the operator of the atomic force microscopy (AFM) to indicate the particle distance and dimensions.

The nanopolymer AFM results allowed morphology at the micro-level (Figures 6A–C). As shown in Figure 6C, its length and width were with a cross-sectional profile in which interchange of heterogeneous wrinkles was visible (Mansouri et al., 2013).

### Extraction and Identification

**FIGURE 7:**
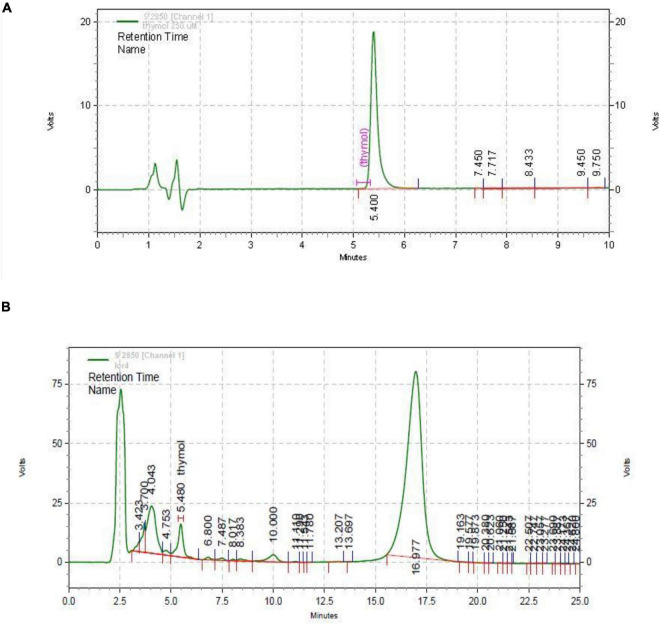
HPLC chromatogram of thymol standard (A) and *Thymbra spicata* extract (B). HPLC conditions were the same.

The HPLC chromatogram of thymol was obtained (retention time = 5.400 min and wavelength = 352 nm). The standard chromatogram of *T. spicata* extract was similar to that of standard thymol in 5.480 min (in the same conditions, Figures 7A,B). The quantitative analysis revealed that thymol was 3.65 mg/g of *T. spicata* extract. The method was calibrated with the linear curve (R2 = 0.9989, *y* = 187,938 × –3,896.03). LOD and LOQ were 52.09 and 173.63, respectively.

### Load and Release Evaluation

The loading and release capacity of thymol and thymol in the extract was evaluated by HPLC. The loading rate of thymol and extract was estimated at 55 ± 3.2% and 48 ± 2.6%, respectively, (*n* = 3).

**FIGURE 8:**
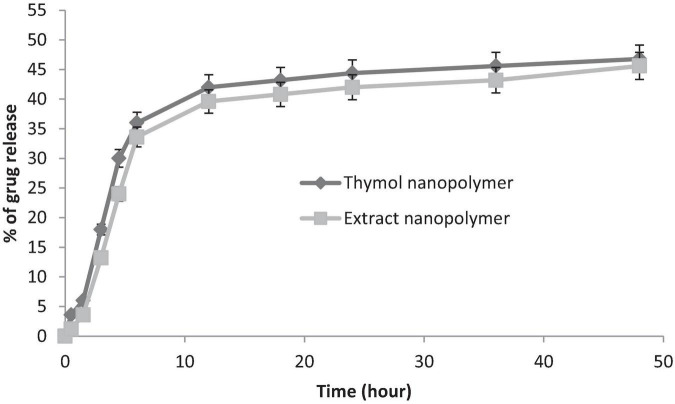
*In vitro* release of thymol and extract-loaded nanopolymer in distilled water.

Thymol and extract were released in two phases (fast and slow) of the nanopolymer. Near to 71% of loaded thymol and 68% of loaded extract in the first 12 h and the remaining 90–92% to 48 h were released at a lower rate (Figure 8).

### Mortality Determination

LD_50_ values of the *T. spicata* extract, thymol, extract nanopolymer, and thymol nanopolymer were 975, 580, 1,250, and 650 mg/kg, within 72 h after administration, respectively. No mortality was observed in the treatment range used in this study.

### Effect of the *Thymbra spicata* Extract, Thymol, Extract Nanopolymer, and Thymol Nanopolymer on the Naloxone-Induced Jumping in Morphine-Dependent Rats

**FIGURE 9:**
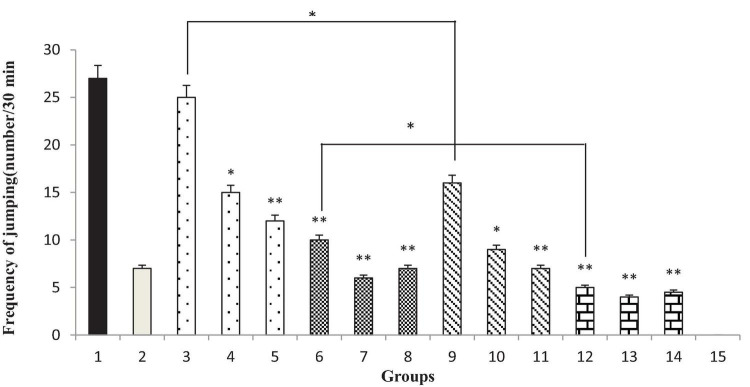
The jumping frequency (number of jumping/30 min) in rats. Group 1; morphine-dependent rats (control). Group 2; morphine-dependent rats and coadministration of clonidine hydrochloride (0.2 mg/kg i.p.) (positive control). Groups 3, 4, and 5; morphine-dependent rats and the coadministration of 100, 200, and 300 mg/kg *Thymbra spicata* extract, respectively. Groups 6, 7, and 8; morphine-dependent rats and coadministration of 30, 60, and 90 mg/kg thymol, respectively. Groups 9, 10, and 11; morphine-dependent rats and coadministration of 100, 200, and 300 mg/kg extract nanopolymer, respectively. Groups 12, 13, and 14; morphine-dependent rats and coadministration of 30, 60, and 90 mg/kg thymol nanopolymer, respectively. Group 15; non-dependent rats (saline). Data are expressed as mean ± SEM. *P < 0.01 vs. control, ^**^P < 0.001 vs. control.

Administration of 200 and 300 mg/kg of *T. spicata* extract and extract nanopolymer (*p* < 0.05 vs. control and *p* < 0.001 vs. control, respectively) and all doses of thymol and thymol nanopolymer (30, 60, and 90 mg/kg) decreased the jumps number in morphine-dependent rats significantly (*p* < 0.001 vs. control) precipitated by naloxone administration (5 mg/kg, i.p.) after the morphine last dose. The effect of *T. spicata* extract and extract nanopolymer was dose-dependent. Clonidine also reduced the jumps number as expected (Figure 9).

### Effect of the *Thymbra spicata* Extract, Thymol, Extract Nanopolymer, and Thymol Nanopolymer on the Naloxone-Induced Rearing in Morphine-Dependent Rats

**FIGURE 10:**
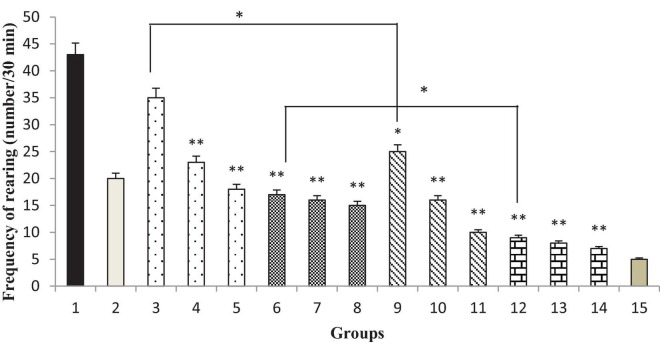
The frequency (number of rearing/30 min) of rearing in rats. Group 1; morphine-dependent rats (control). Group 2; morphine-dependent rats and coadministration of clonidine hydrochloride (0.2 mg/kg i.p.) (positive control). Groups 3, 4, and 5; morphine-dependent rats and the coadministration of 100, 200, and 300 mg/kg *Thymbra spicata* extract, respectively. Groups 6, 7, and 8; morphine-dependent rats and coadministration of 30, 60, and 90 mg/kg thymol, respectively. Groups 9, 10, and 11; morphine-dependent rats and coadministration of 100, 200, and 300 mg/kg extract nanopolymer, respectively. Groups 12, 13, and 14; morphine-dependent rats and coadministration of 30, 60, and 90 mg/kg thymol nanopolymer, respectively. Group 15; non-dependent rats (saline). Data are expressed as mean ± SEM. *P < 0.01 vs. control, ^**^P < 0.001 vs. control.

*Thymbra spicata* extracts and extracts nanopolymer at different doses (200 and 300 mg/kg) and thymol and thymol nanopolymer (30, 60, and 90 mg/kg) decreased the rearing in morphine-dependent rats precipitated by administration of naloxone (5 mg/kg, i.p.) after the last dose of morphine (*p* < 0.001 vs. control). The effect of *T. spicata* extract, thymol, extract nanopolymer, and thymol nanopolymer was dose-dependent. As expected, clonidine reduced the rearing number in animals (Figure 10).

### Effect of the *Thymbra spicata* Extract, Thymol, Extract Nanopolymer, and Thymol Nanopolymer on the Naloxone-Induced Teeth Chattering in Morphine-Dependent Rats

**FIGURE 11:**
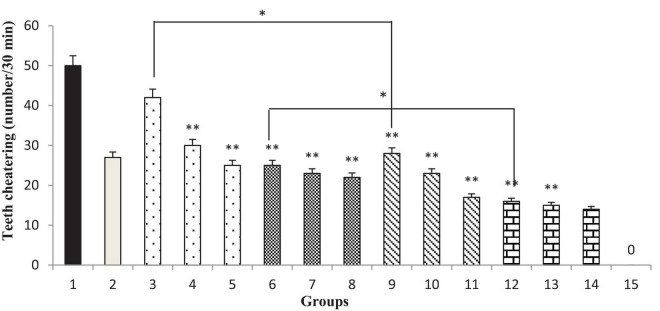
The frequency (number of teeth chattering/30 min) of teeth chattering in rats. Group 1; morphine-dependent rats (control). Group 2; morphine-dependent rats and coadministration of clonidine hydrochloride (0.2 mg/kg i.p.) (positive control). Groups 3, 4, and 5; morphine-dependent rats and the coadministration of 100, 200, and 300 mg/kg *T. spicata* extract, respectively. Groups 6, 7, and 8; morphine-dependent rats and coadministration of 30, 60, and 90 mg/kg thymol, respectively. Groups 9, 10, and 11; morphine-dependent rats and coadministration of 100, 200, and 300 mg/kg extract nanopolymer, respectively. Groups 12, 13, and 14; morphine-dependent rats and coadministration of 30, 60, and 90 mg/kg thymol nanopolymer, respectively. Group 15; non-dependent rats (saline). Data are expressed as mean ± SEM. * *P* < 0.01 vs. control, ^**^P < 0.001 vs. control.

Different doses of the *T. spicata* extract and extract nanopolymer (200 and 300 mg/kg) and thymol and thymol nanopolymer (30, 60, and 90 mg/kg) morphine-dependent rats decreased the teeth chattering in precipitated by administration of naloxone (5 mg/kg, i.p.) after the morphine last dose (*p* < 0.001 vs. control). The effect of *T. spicata* extract, thymol, extract nanopolymer, and thymol nanopolymer was dose-dependent. Also, clonidine reduced the teeth chattering number (Figure 11).

## Discussion

This study showed that the design of drug delivery systems and the production of nanodrugs of *T. spicata* and its bioactive component thymol can be used more effectively in reducing the symptoms of a morphine withdrawal syndrome in rats. In this regard, to reduce the adverse effects, the researchers are now focusing on drug delivery systems. Thymol, a terpenoids structure, which is a bioactive component of some medicinal plants is used for a variety of disorders and diseases (Alvarez Echazu et al., 2017).

Various nanoparticles have been used for many research (Byrne et al., 2008). Recent research has focused on using hyperbranched polymers to increase efficiency due to particular characteristics such as lots of terminal groups, low viscosity, and high solubility (Kolhe et al., 2003; Zhang et al., 2010). They have a similar design to dendrimers, but they are more easily synthesized (Rokicki et al., 2005; Zhu et al., 2006; Wang et al., 2011). In addition, to arrange their compatibility, solubility, chemical recognizability, and reactivity, hyperbranched polymers end-groups can be modified easily (Seiler, 2006). Due to characteristics, namely, excellent biocompatibility and small size, nanoparticles are easily fluid in the bloodstream to increase the likelihood of cell receptors binding (Dulak et al., 2008). In the present study, nanopolymer was synthesized with high biocompatibility and solubility that use for biomedical applications. It has some branches used for loading drugs thymol and extracts as a drug delivery system and all interactions between thymol and nanopolymer were non-covalent. It has been documented that nanopolymer through various mechanisms, such as direct diffusion can penetrate the cell (Meissner, 1986; Verma et al., 2008).

In this study, to get nanopolymer structure, different analytical techniques have been used. The nanopolymer FT–IR spectrum demonstrated carbon derived from the carbonyl group at 1,738 cm^–1^ and a hydroxyl group at 3,314 cm^–1^. The NMR spectrums showed 5 types of hydrogens and 9 types of carbon. The molecular weight of the nanopolymer was obtained by GPC technique around 5,238 g/mol and by DLS tests, the hydrodynamic diameter of the nanopolymer was approximately 75 nm. AFM results showed that nanopolymer was a natural microporous and mesoporous material with polymodal pore size distribution.

Nanopolymer loading capacity was evaluated by HPLC. Based on this analysis, the loading rate of thymol and extract was estimated at 55 ± 3.2% and 48 ± 2.6%, respectively. It has been shown that thymol chitosan nanopolymer had a loading capacity of about 2.5% with antibacterial effects (Medina et al., 2019). But, similar to our study, thymol nanospheres loading was reported to be about 43% (Wattanasatcha et al., 2012).

In this study, it was documented that coadministration of *T. spicata* extract, thymol, extract nanopolymer, and thymol nanopolymer with morphine reduced the severity of the signs of morphine withdrawal syndrome induced by naloxone. These findings demonstrated that the extract and thymol nanopolymer were able to reduce the symptoms of morphine withdrawal syndrome. The effects observed with extract nanopolymer, thymol nanopolymer, and clonidine in reducing the symptoms of morphine withdrawal syndrome were in one direction. Therefore, one of the mechanisms of action of the nanopolymers may be similar to clonidine. Also, the results showed that increasing the dose of *T. spicata* extract, thymol, extract nanopolymer, and thymol nanopolymer decreased the frequency of the signs of withdrawal syndrome, namely, jumping, teeth chattering, and rearing. It has been reported that some supplements and drugs such as magnesium, beta-carbolines, and midazolam reduce the symptoms of withdrawal in the rat (Aricioglu-Kartal and Uzbay, 1997). Moreover, for the management of heroin or methadone withdrawal, clonidine and lofexidine are more effective than placebo (Gowing et al., 2014). Recently, the use of medicinal plants to improve the symptoms of morphine withdrawal has been considered.

It has been reported that some medicinal plants, such as *Zingiber officinale, Glycyrrhiza glabra*, and *Ziziphus jujube* relieve morphine withdrawal symptoms (Doosti et al., 2013). *Thymus* species are the medicinal plants whose leaves and flowering parts are widely used as a tonic, antiseptic, antitussive, and carminative (Ghasemi Pirbalouti, 2009; Jahani et al., 2013; Bakhshi et al., 2014) and also used in pharmaceutical, cosmetic, and preservation of several food products (Surburg and Panten, 2006). The medicinal plant *Zataria multiflora* and *Thymus vulgaris*, thymol-containing, reversed the effects of naloxone due to their analgesic effects (Hosseinzadeh et al., 2000; Taherian et al., 2009). Also, *Thymus daenensis* extract and essential oil attenuate morphine withdrawal behaviors in mice (Khodayar et al., 2014). However, no study has been investigated the effects of the *T. spicata* extract, extract nanopolymer, and thymol nanopolymer on morphine withdrawal syndrome in rats.

Although the *Thymus vulgaris* and its main constituents (thymol and carvacrol) have reduced the symptoms of experimental autoimmune encephalomyelitis in an animal model (Mahmoodi et al., 2019), and thymol has anti-Alzheimer’s (Azizi et al., 2012), antiseizure (Sancheti et al., 2014), antidepressant (Deng et al., 2015), and nAch receptor activity (Sammi et al., 2017), but the effectiveness of *T. spicata* on the nervous system was not observed.

It was also found that thymol treatment increased the Aβ protein levels (Azizi et al., 2012) and the prolonged onset of myoclonic jerk and glutathione levels (Sancheti et al., 2014), and decreased oxidative stress (Lee et al., 2015), norepinephrine in the hippocampus (Deng et al., 2015), and synaptic Ach levels (Sammi et al., 2017).

Our results demonstrate that the ameliorative effects of *T. spicata* extract, thymol, extract nanopolymer, and thymol nanopolymer against withdrawal syndrome by naloxone challenge may be modulated by α2 receptor or PKA pathway in rats. However, extract nanopolymer, and thymol nanopolymer alleviate the morphine withdrawal syndrome more than the total *T. spicata* extract and thymol.

LD_50_ of the *T. spicata* extract, thymol, extract nanopolymer, and thymol nanopolymer were 975, 580, 1,250, and 650 mg/kg, respectively. It has been documented that agents with LD_50_ values in the range of 50–500 and 500–5,000 mg/kg showed moderately and slightly toxic, respectively (Ruiz et al., 2012). By our studies, previous studies have shown that LD_50_ of thymol and thymol nanopolymer were 435 and 583 mg/kg, respectively (Karimi et al., 2019), and other studies have shown that LD_50_ of thymol was 980 mg/kg (Jamil et al., 2010).

## Conclusion

Our results suggest that *T. spicata* extract, thymol, extract nanopolymer, and thymol nanopolymer can reduce the narcotic drugs withdrawal symptoms, and its reducing effect is equivalent to clonidine and may have the potential of narcotic addiction treatment. The efficacy of thymol nanopolymer was greater than the others, and since it affected equivalent to clonidine, its pharmacological mechanism of action is likely to be similar.

## Data Availability Statement

The datasets presented in this study can be found in online repositories. The names of the repository/repositories and accession number(s) can be found in the article/supplementary material.

## Ethics Statement

The animal study was reviewed and approved by the Ilam University of Medical Sciences.

## Author Contributions

All authors listed have made a substantial, direct, and intellectual contribution to the work, and approved it for publication.

## Conflict of Interest

The authors declare that the research was conducted in the absence of any commercial or financial relationships that could be construed as a potential conflict of interest.

## Publisher’s Note

All claims expressed in this article are solely those of the authors and do not necessarily represent those of their affiliated organizations, or those of the publisher, the editors and the reviewers. Any product that may be evaluated in this article, or claim that may be made by its manufacturer, is not guaranteed or endorsed by the publisher.
